# Evaluation of the Bacterial Diversity in the World’s Deepest Cave—Veryovkina, Arabika Massif, Western Caucasus

**DOI:** 10.3390/microorganisms14020368

**Published:** 2026-02-04

**Authors:** Yordan Hodzhev, Violeta Zhelyazkova, Nia Toshkova, Anna S. Barashkova, Borislava Tsafarova, Stefan Panaiotov, Pavel Stoev

**Affiliations:** 1 Department of Natural Sciences, New Bulgarian University, 1618 Sofia, Bulgaria; 2 National Museum of Natural History, Bulgarian Academy of Sciences, 1000 Sofia, Bulgaria; violeta.lyubomirova@gmail.com (V.Z.); niyatoshkova@gmail.com (N.T.); pavel.e.stoev@gmail.com (P.S.); 3 Shemyakin-Ovchinnikov Institute of Bioorganic Chemistry, Russian Academy of Sciences (RAS), ul. Miklukho-Maklaya, 16/10, 117997 Moscow, Russia; barashkova.an@gmail.com; 4 All-Russian Institute of Plant Protection, 196608 Saint Petersburg, Russia; 5 National Center of Infectious and Parasitic Diseases, 1504 Sofia, Bulgaria; b.tsafarova@ncipd.org; 6 Center of Competence “Immunopathogen”, Y. Sakazov 26, 1504 Sofia, Bulgaria

**Keywords:** cave microbiota, Veryovkina Cave, 16S rRNA amplicon sequencing

## Abstract

Veryovkina Cave is the world’s deepest known cave (2212 m deep). It is located in the Arabika Massif of Gagra Mountain in the Western Caucasus. Its microbiome remains unknown because of difficulties in access. Ten sediment samples were collected at vertical depths ranging from 300 m to 2204 m; they varied by substrate type, moisture content, and visitor accessibility. Total microbial DNA was isolated, and 16S ribosomal gene metabarcoding was applied for taxonomic identification. Seven samples showed reliable content, whereas three samples indicated no recoverable reads. Proteobacteria, Acidobacteria, and Actinobacteria were the most abundant phyla in total. Depth stratification of microbiota showed that (1) shallow wet clays were dominated by Acidimicrobia and Actinobacteria; (2) mid-depth wet clays showed the highest abundance of Nitrospira, Betaproteobacteria, and Vicinamibacter; and (3) deep, dry substrates were dominated by Thermoleophilia and Rubrobacteria. Multivariate analyses showed that substrate type and moisture tended to explain more variation in microbial abundance than depth or human activity. We demonstrate the presence of distinct ecological niches within the cave ecosystem, which emphasizes the role of local conditions in shaping microbial diversity.

## 1. Introduction

Caves are specific habitats characterized by the absence of light, niche isolation, oligotrophy, diverse humidity, and strong depth gradients. Cave microorganisms are essential drivers of the ecosystem [1]. Due to limited energy sources, microbiota employ chemolithoautotrophy and the decomposition of scarce organic matter [2]. Cave bacteria influence both cave geochemistry and the formation of secondary mineral structures, such as moonmilk deposits and speleothems [3,4]. Typical cave microbiota include Acidobacteria, Actinobacteria, Pseudomonadetes, Chloroflexi, and Planctomycetes. These microbial groups drive nitrogen cycling, sulfur oxidation, and mineral precipitation [2,5,6].

Ecological factors that modulate the composition of cave microbial communities have been widely investigated. Some of these are natural, such as the sample type (e.g., air, water, rock, or sediment) [7], substrate geochemistry [1,8], nutrient input [9], or the presence of bats [10]. Others can be anthropogenic, such as exploration [11], tourism [12], or artificial lighting [13], with considerable attention being paid to caves decorated with prehistoric paintings, such as Lascaux in France [14,15]. Despite reports of some common taxa, most research points to a high level of endemism, in terms of both animal and microbial life [16]. Caves represent a unique model for multiple interacting environmental gradients that collectively influence the microbial community structure.

Moving away from the surface, light, temperature, organic carbon input, and human visitation typically decrease, while isolation and mineral exposure increase [17,18]. Kieraite-Aleksandrova et al. (2015) [19] studied the microbiota of the second-deepest cave in the world—Krubera-Voronja Cave (2199 m deep, Gagra Mountain, Arabika Massif, Western Caucasus). They described over 20 bacterial phyla [19]. Shallow zones (220–230 m) showed a mixture of a large number of relatively evenly distributed microbial taxa, whereas the deepest sites (~1200 m) were dominated by Actinobacteria, along with other, less abundant oligotrophic phyla. Krubera–Voronja Cave has been visited by expeditions for decades. It is reasonable to assume that this frequent human activity has influenced the microbial community composition by introducing surface microbes and increasing organic inputs.

Therefore, the exploration of Veryovkina Cave, which has far more difficult access and whose deepest galleries were discovered only recently, provides an opportunity to study relatively conserved microbial communities. Veryovkina Cave is located approximately 20 kilometers away from Krubera–Voronja Cave. It reaches 2212 m below the surface (or 2204 when our study was conducted) [19,20,21].

The aim of this study is to address the lack of information about microbial life in the deepest karst formations. We investigate how the bacterial community composition varies with environmental factors inside Veryovkina Cave. Specifically, we ask the following questions: (1) How does microbial abundance change with vertical depth, substrate type, and moisture gradients? (2) Which taxa dominate the cave ecosystem and what are the distinct ecological niches? (3) Do these communities suggest functional differences across habitats? We hypothesize that local microenvironmental factors, such as the sediment type and moisture content, exert a stronger influence on bacterial diversity and structure than depth alone. We explore microbial diversity at ten sites spanning from 300 m to 2204 m in depth (1904 m vertical range) and various sediment types, moisture content levels, and degrees of visitor access [17].

## 2. Materials and Methods

### 2.1. Sample Collection

**Figure 1:**
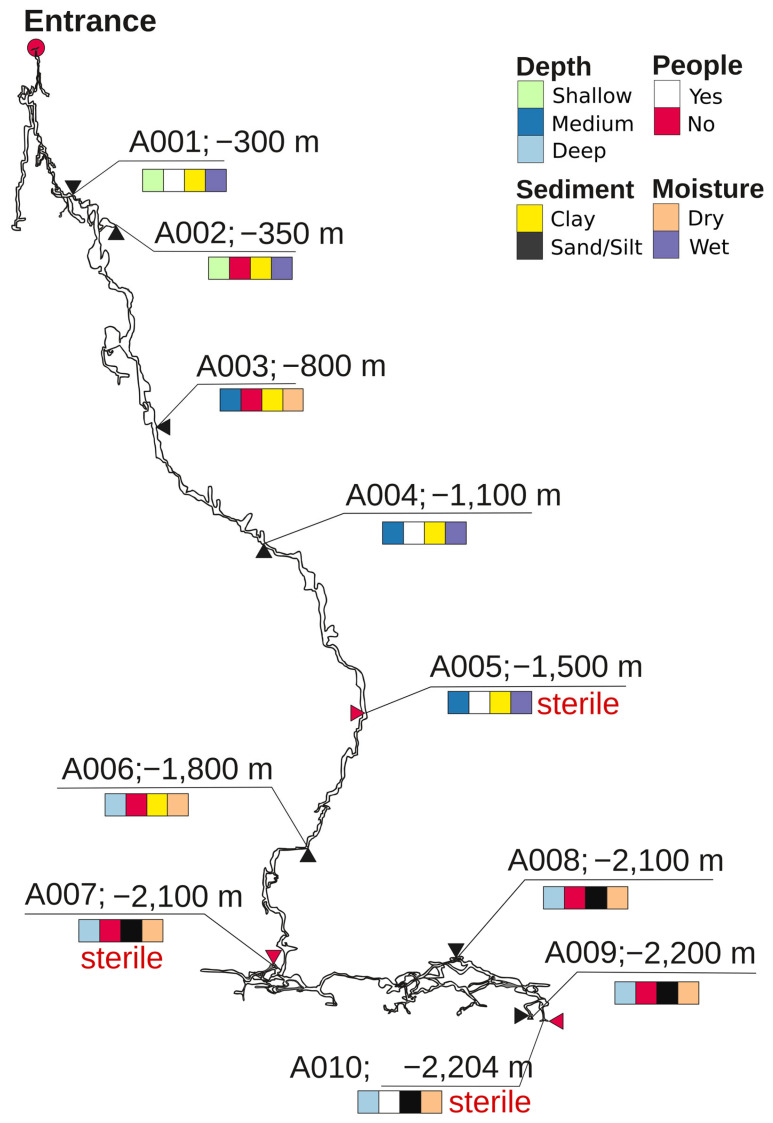
Depth map of Veryovkina Cave, showing sampling sites A001–A010. Substrate type, water content, and human visitation are color-coded. Sites indicated as sterile are shown with red arrows and red inscription “sterile” (A005, A007, and A010).

Samples were collected by organized members of the Perovo Speleo caving club in August 2019. Figure 1 illustrates the sampling sites located along the entire vertical profile of the cave, spanning from the entrance to its maximum depth of −2204 m, with a length of approximately 17.5 km. Additionally, they represented various substrates (clay, sand, silt), water content levels (wet vs. dry), and human disturbances (yes vs. no). From each site, substrate was collected using a sterile spoon and placed in a 15 mL tube filled with 70% ethanol, because this solution is particularly effective in the inactivation of microbial nucleases [20]. Metadata for each sample are presented in Table 1.

### 2.2. DNA Isolation and Amplicon Sequencing

A standard soil kit (DNeasy PowerSoil Pro Kit, Qiagen, Germany) was used for DNA extraction. The mean microbial DNA concentration was approximately 1.8 ± 1.4 ng/μL, with an average A260/A280 purity ratio of 1.72 ± 0.08. V3-V4 hypervariable regions of the 16S rRNA gene were amplified with universal primers 341F and 805R, producing amplicons of 465 bp. Bidirectional sequencing of 2 × 300 bp paired-end reads was performed on an Illumina MiSeq system. A negative control was used to control for microbiological contamination—a mixture of used reagents without biological material. Sequencing data have been deposited in the NCBI Sequence Read Archive under BioProject accession number PRJNA1221685.

### 2.3. Data Processing and Normalization

Taxonomic identification of the seven sediment samples was performed using the EzBioCloud platform (v. 2024) [22]. This is a comprehensive and quality-controlled prokaryotic taxonomy database and identification pipeline that integrates 16S rRNA gene and whole-genome reference sequences [23]. Within EzBioCloud, we applied the standard identification pipeline whereby dereplicated sequences are queried via the VSEARCH tool (v. 2.29.3:) against the reference database and assignments are made using similarity thresholds (for example, ≥97% for species level), as described in the supporting documentation. After the subtraction of the bacterial reads from the negative control sample, abundance tables were processed using the MicrobiomeAnalyst (v. 2.0) web-based platform for microbiome data analysis, visualization, and meta-analysis [24,25,26]. In MicrobiomeAnalyst, we uploaded operational taxonomic unit (OTU) count tables derived from the EzBioCloud assignments. For each sample, the raw OTU counts were calculated at the phylum and class levels. Rare or unclassified groups were kept but not interpreted. The overall cave abundance profile was calculated by summing across all samples.

### 2.4. Data Analyses

Community differences across sampling sites were visualized using heatmaps of relative taxon abundances. For alpha diversity (within-sample variation, Fischer’s coefficient) and beta diversity (between-sample variation) analyses, we used the MicrobiomeAnalyst platform [26]. In the assessment of beta diversity, a principal coordinate analysis (PCoA) was performed using the Bray–Curtis index [21]. For hypothesis testing, we computed the distance matrix and performed a permutational analysis of variance (PERMANOVA) [27]. Univariate group statistics were performed via the Kruskal–Wallis test.

## 3. Results

### 3.1. Sample Description

Figure 1 shows a vertical map of the Veryovkina Cave and the richness and distribution of the substrate types, water content, and human access. Samples from shallow sites A001 and A002 were collected from wet clay substrates. Site A001 was visited by speleologists, whereas A002 was unvisited. Sample A003 was collected from dry clay from a non-visited site. Samples A004 and A005 were collected from wet clay from an isolated location. Deeper samples included dry samples from unvisited sites and consisted of clay (A006) and sand (A007, A008, and A009). In contrast, the deepest sample—A010—was collected from dry silt at a visited location.

### 3.2. Overview of Bacterial Taxonomic Diversity in Veryovkina Cave

**Figure 2:**
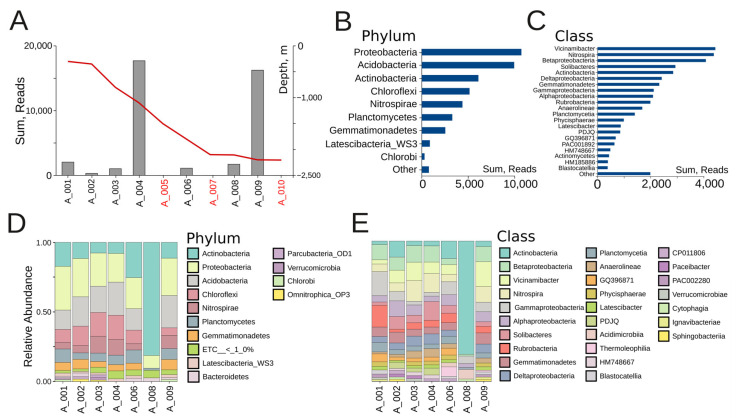
(**A**) Microbial abundance of Veryovkina Cave. The red line indicates the depth progression through the cave in meters. Depth axis is shown on the right side of the panel (sterile locations are depicted in red). (**B**) Overall microbial abundance across bacterial phyla, summed across all samples. (**C**) Overall microbial abundance across bacterial classes, summed across all samples. (**D**) Phylum-level relative abundance. (**E**) Class-level relative abundance.

We managed to isolate DNA from 7 out of the 10 samples from Veryovkina Cave (Figure 1). Across the positive samples, DNA quantities were low, reflecting scant microbial life. Bacterial richness significantly varied between sites (Figure 2). The two locations with the richest microbiota were A004 and A009, each yielding more than 15,000 reads (Figure 2A).

The most abundant bacterial phyla were Proteobacteria (~10,000 reads), Acidobacteria (~9500 reads), and Actinobacteria (~6000 reads), followed by Chloroflexi and Nitrospirae. Together, the first three phyla accounted for more than 60% of all reads (Figure 2B). The dominant classes were Vicinamibacter, Nitrospira, and Betaproteobacteria, each contributing more than 4000 reads and together forming the functional backbone of nitrogen and carbon cycling within the cave (Figure 2C). These were followed by Solibacteres and Actinobacteria, both exceeding 2800 reads, and Deltaproteobacteria and Gemmatimonadetes, each present with over 2000 reads. At intermediate abundance levels, Gammaproteobacteria, Alphaproteobacteria, Rubrobacteria, Anaerolineae, and Planctomycetia were detected, with between 1500 and 2000 reads each. The lowest-abundance classes included Physcisphaerae, Latescibacter, PDJQ, GQ396871, PAC001892, HM748667, Actinomycetes, HM185886, and Blastocatellia, with most contributing fewer than 1000 reads each (Figure 2C).

At the individual sample level, both shared trends and differences among sites could be observed. Across most samples, the three dominant phyla were Proteobacteria, Acidobacteria, and Actinobacteria, together contributing more than half of the relative abundance (Figure 2D). Samples A001, A002, and A004 displayed relatively balanced communities, with Proteobacteria and Acidobacteria in comparable proportions, alongside Chloroflexi and Nitrospirae. The shallow clay sample A001, under human influence, was characterized by elevated Acidimicrobia and Actinobacteria—taxa that are tolerant to disturbances—alongside Cytophagia, which likely reflects surface-derived organic inputs. A002, which was also a shallow clay but undisturbed, shared the broad Proteobacterial background yet stood out through the enrichment of Acidimicrobia and PAC002280. In contrast, A004, a moist mid-depth clay under human contact, harbored the richest community, with balanced contributions from Nitrospira, Betaproteobacteria, Planctomycetia, and Vicinamibacter. A003 and A006 also showed a mix of the same phyla, albeit with higher relative contributions of Chloroflexi and Nitrospirae. A003, a medium-depth dry clay, showed intermediate diversity dominated by Rubrobacteria and Gemmatimonadetes. Sample A006 (a deep dry clay) was enriched in Thermoleophilia, Anaerolineae, and Rubrobacteria. In contrast, A008 was almost completely dominated by Actinobacteria. The sandy sample A008 hosted the lowest richness, dominated by Rubrobacteria and Thermoleophilia, characteristic of narrow oligotrophic communities. Sample A009 showed high proportions of Acidobacteria and Proteobacteria. A009 also showed high abundances of Nitrospira, Vicinamibacter, and Betaproteobacteria.

At the class level, Figure 2E provides a more detailed view of the community structure, highlighting individual differences among sampling sites. As at the phylum level, the dominant classes across most sites included Actinobacteria, Betaproteobacteria, Vicinamibacter, Nitrospira, Gammaproteobacteria, and Alphaproteobacteria, but their proportions varied strongly between locations. For example, A008 was almost exclusively dominated by Actinobacteria, while A004 and A009 retained higher evenness, with notable contributions from Betaproteobacteria, Vicinamibacter, and Nitrospira. In contrast, A001 was enriched in Gammaproteobacteria relative to samples from deeper sites. Importantly, class-level resolution revealed several specific bacterial groups. These included Ignavibacteriae, Sphingobacteria, Cytophagia, Acidimicrobiia, Thermoleophilia, Blastocatellia, and Verrucomicrobiae, all of which appeared in minor proportions across certain samples. This demonstrates the presence of specialized lineages that may occupy micro-niches in specific substrates (e.g., wet clays or sand deposits). Classes such as Anaerolineae and Deltaproteobacteria also emerged more distinctly at this resolution.

At the family level, Figure S1A reveals an even higher degree of taxonomic diversification compared to the phylum and class profiles. Across all sampling sites, a total of over 30 prominent families were detected, although many were found to be uncultured or poorly described lineages designated by environmental codes (e.g., PAC, EU, AM, DQ series). This highlights both the richness of Veryovkina Cave’s microbiota and the large proportion of novel taxa that still are not formally described. Several families stood out as ecologically significant. Cytophagaceae were strongly dominant in A008, reflecting the unusual Actinobacteria-/Cytophaga-rich signature of this deep sand site. Vicinamibacteraceae, Solibacteraceae, and Gemmatimonadaceae were abundant across multiple sites. Nitrospiraceae and Planctomycetaceae were consistently present. In addition, families such as Gaiellaceae, Rhodospirillaceae, and Pseudomonadaceae contributed to site-specific profiles, often in shallow or mid-depth clay sediments. In total, only about 10 to 12 families could be confidently identified, including Nitrospiraceae (g. *Nitrospira)*, Pseudomonadaceae (g. *Pseudomonas*), Rhodospirillaceae, Planctomycetaceae, Solibacteraceae, Vicinamibacter, Gemmatimonadaceae, Gaiellaceae, Cytophagaceae, and Bacillaceae. These accounted for only a minor fraction of the total reads, with the remainder classified as uncultured or environmental lineages.

At the genus level (Figure S1B), the community profiles became highly fragmented, with a large proportion of sequences assigned to uncultured or coded lineages (e.g., PAC, EU, AM, GQ clusters). Only a few genera, such as *Nitrospira*, were consistently identifiable across samples, while most reads were distributed among dozens of poorly resolved bins. Therefore, for the purposes of ecological description and comparison across sites, we determined that the class level provided the optimal balance between taxonomic resolution and interpretation.

**Figure 3:**
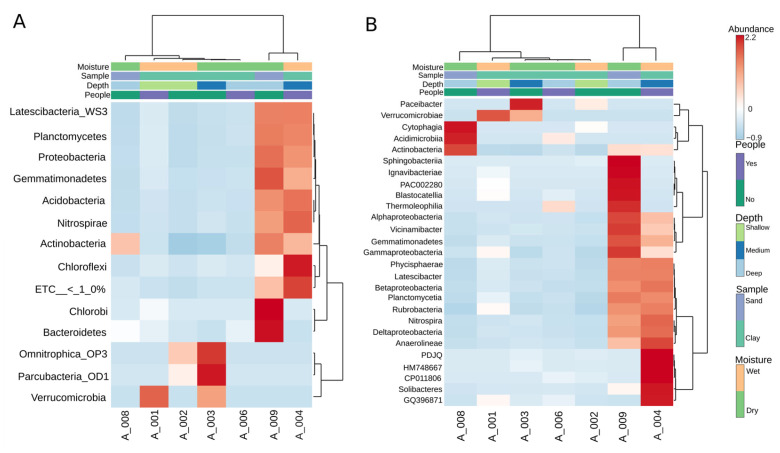
Heatmap analysis of bacterial community composition across cave samples at phylum (**A**) and class (**B**) levels. A Euclidean dendrogram visualizes the hierarchical clustering of both samples and taxa.

As seen in Figure 3, the heatmap analysis revealed three major ecological groupings within the cave communities. Cluster 1 comprised A004 and A009, where mid-depth wet clays and one sandy site were grouped together through their high diversity and balanced contributions from Nitrospira, Betaproteobacteria, and Acidobacteria. Cluster 2 included the shallow wet clays A001 and A002, both dominated by Acidimicrobia and Actinobacteria, which reflect visitation-linked and surface-associated taxa. Cluster 3 encompassed the deep dry sites A006 and A008, enriched in Thermoleophilia, Rubrobacteria, and Anaerolineae, forming oligotrophic end-members with highly specialized and narrow communities. Finally, A003 occupied an intermediate position, bridging shallow and deep clusters through a mixed composition of Rubrobacteria and Gemmatimonadetes, highlighting its transitional character between contrasting ecological states.

### 3.3. Factors Shaping Bacterial Community Structure in Veryovkina Cave

**Figure 4:**
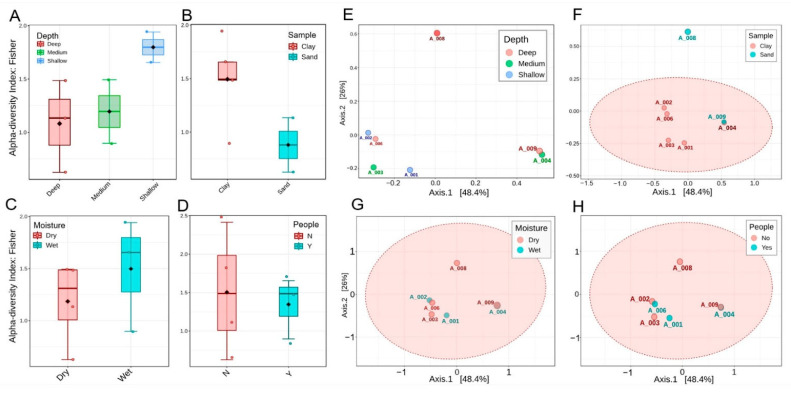
Alpha and beta diversity patterns of bacterial communities across cave sites, calculated at phylum level. (**A**–**D**) Fisher’s alpha diversity index grouped by depth, substrate, moisture, and human disturbance, respectively, showing higher richness in shallow and moist clays compared to deep dry sands. None of these reached statistical significance (Kruskall–Wallis, *p* > 0.5). (**E**–**H**) PCoA plots based on Bray–Curtis distances, colored by depth (**E**), substrate (**F**), moisture (**G**), and human presence (**H**).

The distinct bacterial compositions in our samples could be explained descriptively by several factors, such as depth, sediment type, moisture, and human presence. However, the Kruskal–Wallis test showed no significant differences between groups (*p* > 0.05). Figure 4A shows that shallow sites demonstrated the highest alpha diversity, mid-depth sites displayed intermediate values, and deep sites were more variable, with generally reduced alpha diversity. Figure 4B demonstrates that clay substrates sustain richer communities than sand, which exhibit lower diversity. Figure 4C indicates that wet habitats support greater diversity compared to dry ones, emphasizing the role of moisture in maintaining balanced microbial communities. Figure 4D highlights the effects of human presence, with undisturbed sites showing broader variation and higher richness, whereas disturbed sites are more constrained and less diverse. In the PCoA plots, Figure 4E reveals clear separation by depth, with shallow, medium, and deep samples distributed along the first axis and deep sites A008 and A009 positioned furthest apart. Figure 4F confirms the influence of the substrate, with clay sites forming a coherent group and sandy samples emerging as distinct outliers. Figure 4G compares moisture categories, showing general overlap but with A008 remaining a strong outlier under extreme dryness. Finally, Figure 4H illustrates that disturbed and undisturbed sites overlap substantially, suggesting that disturbance is secondary to depth and substrate in structuring communities, although the distinct separation of A008 persists across all comparisons. Although the first two PCoA axes explained a large proportion of the variance (PC1 = 48.4%, PC2 = 28.0%), the PERMANOVA did not detect significant differences between groups (*p* > 0.05). This indicates either that the major sources of variation in community structure were not sufficiently aligned with the tested environmental variables or that the small number of analyzed samples limited the power of statistical tests.

## 4. Discussion

The present study is the first to examine the microbial diversity in Veryovkina Cave, the deepest cave in the world. Our data reveal both general trends across samples and location-specific bacterial communities. For example, at the phylum and class levels, several groups are widely represented throughout the cave system. Proteobacteria, Actinobacteria, Acidobacteria (Solibacteres, Vicinamibacter, Blastocatellia), and Nitrospirae (Nitrospira) occur in most samples, albeit in different relative proportions. Some samples are dominated by single classes, such as Actinobacteria (A008) or Gammaproteobacteria (A001), while others (A004, A009) maintain higher diversity. This is consistent with observations from other limestone caves, where oligotrophic guilds sustain primary production and nutrient cycling. Proteobacteria and Acidobacteria often form the core of the cave microbiome, dominating as versatile heterotrophs and chemolithotrophs. Their broad distribution suggests the resilience of the microbiota to factor changes. Betaproteobacteria and Nitrospira are nitrifiers, maintaining the nitrogen cycle, while Deltaproteobacteria contribute to anaerobic respiration via sulfate reduction. Acidobacteria and Solibacteres are typical oligotrophs and play key roles in organic matter degradation. Together, these groups form a microbial network that maintains biogeochemical stability in the absence of photosynthesis.

However, these different habitats should remain interconnected through the potential exchange of microbiota via air, water, and, to some extent, human explorers. A question meriting further investigation is the influence of flooding on Veryovkina’s microbial communities. A particularly large flood was documented during an expedition in the cave in September 2018 that endangered the lives of expedition participants and gained broad media attention. Such events are, in fact, common in vertical alpine caves, which can quickly take up large volumes of rainwater or melting snow in spring. Thus, it would be interesting to study the responses of cave microbiota to such extreme disturbances and test the following alternative hypotheses. On the one hand, it is possible that running water increases the interconnectedness between different parts of the cave by bringing microbes and organics from the surface and the upper parts of the system to its bottom. On the other hand, water can also wash away certain microbes, creating post-disturbance bottlenecks and shaping patchy survivor communities. Such profound impacts of flooding, combined with pollution, have been documented in Pindal Cave in Northern Spain, where a flood induced great alterations in the native microflora [28]. Our current data, however, are not sufficient to disentangle the potential influences of flooding and other environmental factors shaping Veryovkina’s bacterial communities, where the substrate type seems to play the greatest role in our sample composition. This supports the notion that both factors are central to the microbial compositions of cave ecosystems [3,5]. Evidence from the Baeg-nyong moonmilk deposits further supports the role of mineral substrates in structuring communities, with Actinobacteria, Acidobacteria, and Proteobacteria dominating wet versus dry moonmilk niches [25].

To understand the specificity of the bacterial communities in Veryovkina Cave, we compared our results to those from the second-deepest cave in the world, Krubera-Voronja, located in the same mountain range, just 12 km from Veryovkina. In total, 24 phyla were identified in soil samples from Krubera Cave [17], with the core set dominated by Proteobacteria (*Pseudomonas*, *Escherichia*), Actinobacteria (*Propionibacterium*, *Micrococcus*), and Firmicutes. However, while Proteobacteria and Actinobacteria also dominate Veryovkina’s backbone groups, Firmicutes are not as well represented. Krubera samples contained up to 165 genera per sample, while fewer were identified from Veryovkina. This may indicate distinct bacterial richness in the two caves, or it may reflect methodological differences. Furthermore, Krubera’s microbiota were more strongly influenced by depth and human visitation. Actinobacteria dominated the upper parts of the cave, whereas Proteobacteria dominated in the deeper zones. Similarly, the Firmicutes abundance decreased with depth, whereas that of Bacteroidetes increased. These observations are in contrast to those in the neighboring Veryovkina Cave, where the sediment type had a stronger effect than the depth. Importantly, in Krubera Cave, frequent human visits had a significant impact on the microbiome, leading to notable alterations in the microbial composition [17]. In Veryovkina, visitations did not show such an effect because of the significantly lower frequency of expeditions. Additionally, more samples from Kieraite-Aleksandrova’s study were collected from underground basecamps in Krubera, where expedition participants store and consume food and leave their biological waste, thus not only bringing foreign microbes but also changing the local nutrient availability [19].

Studies from other caves around the world also show the roles of different microhabitats, human-influenced or not, in shaping subterranean microbial communities. In Baeg-nyong Cave, South Korea, the microbial profiling of wet and dry moonmilk deposits showed that the community composition differed not only from that of external soils but also between moonmilk types, with Proteobacteria, Actinobacteria, and Acidobacteria as the dominant groups [29]. Moonmilk formation is mediated by bacterial activity through calcium carbonate precipitation [3,4]. In the Shulgan-Tash Cave of Southern Ural, Russia, environmental factors such as humidity, temperature, and sediment type strongly influenced the taxonomic compositions of cave biofilms, i.e., tapestries [30]. Similarly, in the Brestovská Cave of the Western Tatras, Slovakia, soil and sediment samples harbored diverse culturable micromycetes, reflecting the role of substrate heterogeneity in shaping fungal diversity [31]. Multiple authors have also addressed human influences on cave microbial systems, but with contradicting results. For example, Shapiro and Pringle (2010) found the highest fungal diversity in soil samples from rarely visited cave sections (negative relationship) [32], while Mammola et al. (2017) [33] found a positive correlation, and Piano et al. (2023) and Bercea et al. (2019) reported different trends for specific fungal and bacterial groups [12,34].

## 5. Conclusions and Future Work

In our study, we discovered both common patterns and distinct features of the bacterial communities in Veryovkina Cave. Although a number of taxa were shared between sampling locations, most samples showed specific bacterial profiles, underlining the importance of different microhabitats within the cave system, where the substrate composition and water content seemed to play a more important role than depth. However, to fully understand microbial life in the deepest places on Earth, we would need to analyze a larger number of samples, combine taxonomic and functional genetic analyses, collect more data on substrate geochemistry, and compare results from different caves obtained with the same methodology. It would also be interesting to compare samples collected before and immediately after flooding events to explore the resilience of subterranean microbial communities to extreme disturbances. Given the difficulties in obtaining and transporting samples from the planet’s most harsh and remote environments, our study is a small but useful step toward answering such questions.

## Figures and Tables

**Table 1:** Sampling sites in Veryovkina Cave and their environmental characteristics.

Sample	Depth (m)	Depth Category	Water Content	Sediment	Human Disturbance	Sampling Site
A_001	−300	Shallow	Wet	Aerosol clay	Yes	Basecamp with hole
A_002	−350	Shallow	Wet	Clay	No	Over the “Oiylia pass”—newly discovered
A_003	−800	Medium	Dry	Clay	No	Stone collapse “suitcase warehouse”—disrupted calcite crust
A_004	−1100	Medium	Wet	Pink clay	Yes	Pink meander—place of the least human impact
A_005	−1500	Medium	Wet	Clay	Yes	Poltorashka—meander at the bottom of the pit
A_006	−1800	Deep	Dry	Clay with fungi	No	Wall after the half-syphon bypass in front of the inflow
A_007	−2100	Deep	Dry	Sand	No *	Labyrinth over way to the cave bottom
A_008	−2100	Deep	Dry	Sand	No	Over Stephan Razin’s Lake
A_009	−2200	Deep	Dry	Sand	No *	Captain Nemo’s last stand—sandy “beach” near the bottom syphon, traces of invertebrates
A_010	−2204	Deep	Dry	Silty sediment	Yes	Captain Nemo’s last stand—sandy “beach” near bottom syphon

* The gallery had not been visited after the flood in 2018.

## Data Availability

The original contributions presented in this study are included in the article/Supplementary Materials. Further inquiries can be directed to the corresponding authors. All raw sequencing data generated in this study have been deposited in the NCBI Sequence Read Archive under BioProject accession number PRJNA1221685; see https://www.ncbi.nlm.nih.gov/search/all/?term=PRJNA1221685 (accessed on 26 December 2025).

## Supplementary Materials

The following supporting information can be downloaded at https://www.mdpi.com/article/10.3390/microorganisms14020368/s1: Figure S1: (A) Family-level relative abundance; (B) genus-level relative abundance.
