# Supplementary figures and images for: Evaluation of the Bacterial Diversity in the World’s Deepest Cave—Veryovkina, Arabika Massif, Western Caucasus
Source: Microorganisms. 2026 Feb 4;14(2):368. doi: 10.3390/microorganisms14020368 (PMC12942678; doi:10.3390/microorganisms14020368)

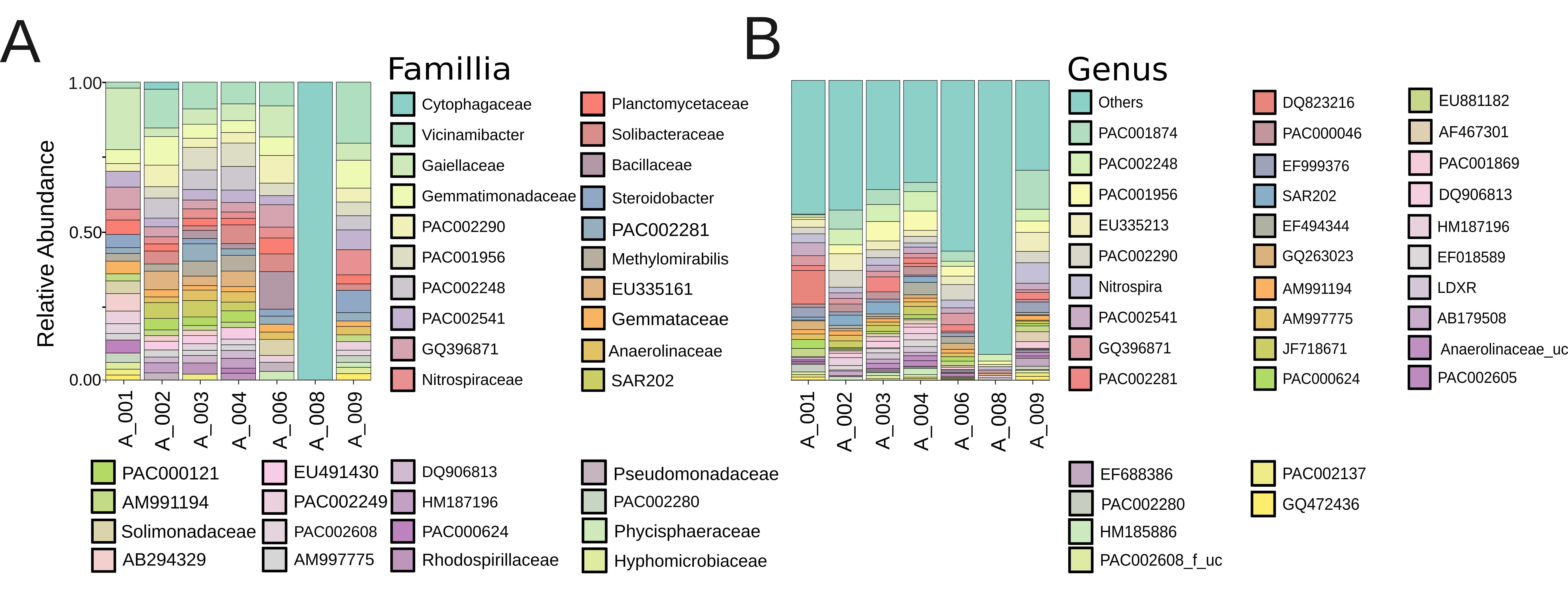

Supplement: Supplementary file 1 [file microorganisms-14-00368-s001.zip › microorganisms-4019863-supplementary.png]
